# A Treatise on Poisons

**Published:** 1830-04-01

**Authors:** 


A TREATISE ON POISONS, in RELATION TO MEDICAL JURISPRUDENCE, PHYSIOLOGY, AND THE PRACTICE OF PHYSIC. By *Robert Christison*, M.D. Professor of Medical Jurisprudence and Police in the University of Edinburgh. 1 Vol. 8vo. pp. 700, price 16s. 1830.

SINCE the beginning of the present century, a third of which is nearly lapsed, only one systematic work on Toxicology has appeared in the English language—and that was a translation from the French of Orfila—and never finished. Two parts of his work were published in 1816, and the translator has the following passage in his preface. "It is to be hoped that his (Orfila's) example will be followed up by some of our own countrymen, possessed of talent and leisure, sufficient for the undertaking. By frequently repeated experiments, there is no doubt but this science may be brought to some degree of perfection." Fourteen years passed away, before one of our countrymen, possessed of the requisite talents, came forward as a systematic Toxicologist—and Dr. Christison is the man. Dr. Paris, indeed, from the circumstance of his dedicating 336 pages of his second volume of Medical Jurisprudence to the subject of Poisons, may be considered as a systematic toxicologist; but still, a seven years' increase of chemical knowledge, and accumulation of jurisprudential facts, and an extension of the investigation to double the space allotted by Dr. Paris, must render the present work, if ably executed, the standard of reference in this important department. That Dr. Christison is well qualified for the task, can hardly be doubted, not only from the many scientific papers which he has published on poisons, but from his appointment to the chair of Medical Jurisprudence in Edinburgh. Dr. Duncan too, who is evidently—indeed avowedly, the reviewer of Dr. Christison's work in the last volume of our venerated northern cotemporary, has given a testimony in favour of the volume before us, which must have great weight with the public at large. "Considering (says Dr. Duncan) Dr. Christison as, in some measure, our professional son, we feel proud to acknowledge that he has effected what we, at no time, were able to perform, and, reversing the original meaning, to say of him, "Sequiturque patrem non passibus æquis." That such a work as the present was a desideratum, cannot be doubted—and that it will prove a standard reference for all classes of practitioners, we decidedly prognosticate. Of such a mass of elementary matters it will not be expected that we should attempt any regular analysis, though we shall select some particular subjects for distinct articles in this journal.

The importance of the study of toxicology is ably stated in a sensible preface. When indeed we look into the daily prints, not one of which is unfurnished with instances of murder, suicide, and accidental poisons—when we consider the interest attached to such catastrophies, and the rigid scrutinies by which they are brought to light—we cannot be blind to the necessity of a comprehensive knowledge of toxicology on the part of the medical practitioner, whatever be his rank or designation. But allowing that he was secure from giving evidence in a court of justice (which he never can be) the science of toxicology is highly important in the common avocations of his profession. It supplies antidotes for the various poisons—(and all medicines are poisons as well as remedies)—it elucidates the laws of the animal economy—and aids him in his inquiries into the action of energetic drugs. Thus to toxicology he owes the knowledge of albumen as an antidote to oxymuriate of mercury and verdigris—of bark against tartrite of antimony—of the alkaline sulphates against acetate of lead—of the earthy and alkaline chlorides for sulphuret of potass—of ammonia and chlorine against hydrocyanic acid, &c.

The laws of absorption have been elucidated by the experiments of the toxicologist, who has introduced many valuable active medicines into the practice of the physician, as strychnia for example. Still it is in medical Jurisprudence that toxicology shews its power and extent to the greatest advantage. In cases of poisoning, the weighty part of the proof must necessarily concentrate in the medical evidence, since it can seldom be drawn, as in other kinds of homicide, from the attendant circumstances of the case. It is fortunate that the resources of the medical witness are generally commensurate with the serious responsibility imposed upon him. By semeiology he distinguishes the symptoms of poisoning from those of natural diseases—by pathology he discriminates the appearances in the dead body—by chemistry he discovers foreign substances of a deleterious nature in the body and elsewhere—and by experimental physiology he determines the value of evidence, from the effects of poisons on the lower animals. To use the words of our author, toxicology "ranges over the whole vast field of medical learning, and draws together, from a variety of quarters, facts and principles which are seldom at any other time viewed in combination. The resources of each science are thus made to try the accuracy and supply the defects of the others; and the whole mass of knowledge is brought to bear in one direction with a force and precision worthy of its objects—the detection of crime, and the vindication of innocence."

Since our author's appointment to the jurisprudential chair in Edinburgh, he has been more extensively employed in the practice of toxicology than perhaps any physician in these islands. In his researches he soon discovered the numerous defects and deficiencies of works on toxicology—defects probably arising from the attention having been too exclusively turned to the means by which particular poisons may be proved to have been the cause of death—whereas the questions which actually occur in medico-legal practice are much more diversified.

At coroners' inquests, the medical man has often to inquire into cases which are really instances of natural death, though construed, from peculiar attendant circumstances, into murder. Hence he has not only to prove poison when actually administered, but be prepared to shew the negative side of the question in a clear light, by proving that death arose from common disease. In these cases it often happens that only a general suspicion of poisoning exists, without any particular poison being indicated—and here the physician is called upon to say whether there is a certainty, probability, or possibility of poisoning, in a *general sense*. These questions, but little adverted to by preceding writers, are dwelt upon at great length by Dr. C. under the head of "Evidence of General Poisoning," of which we shall give a pretty full account presently. On trials before the high Criminal Courts the prisoner is usually charged with administering a particular poison, and also some poison unknown to the prosecutor. In some instances the evidence of the particular poison is merely presumptive—and that not strong, so that the charge is substantially one of poisoning in a general sense. "Convictions have been recently obtained under such charges, where no satisfactory proof existed what poison had been given." Dr. C. differs therefore from the German and French writers on Medical Jurisprudence, who all express an opinion that poisoning can never be completely substantiated, unless the particular poison be found out. It is highly improbable, however, that the evidence of general poisoning, drawn solely from a medical source, will ever convict an individual. There must be strong moral or circumstantial evidence to corroborate the medical.

In the chemical search after individual poisons Dr. C. has made it an invariable object to select processes which are, at the same time, delicate, conclusive, and easily managed by the inexperienced. This is a great recommendation of the work, especially when it is remembered that there are very few of these processes whose accuracy he cannot vouch for, in consequence of frequent trials made under the most difficult circumstances. Some of the processes are new, and, he thinks, preferable to any previously known. These are chiefly the processes for arsenic, mercury, and opium. Some improvements have also been suggested in the mode of detecting copper, lead, zinc, and oxalic acid. Dr. C. has made some experimental researches on the action of various waters on lead, the results of which are interesting.*

---

* Many of the more important tests will be given in insulated articles of our Periscope.

The work is divided into two parts—the first (containing 80 pages) on general poisoning—the second (more than 600 pages) on individual poisons. Of the first part we shall attempt an analysis in this article.

### PART I. ON GENERAL POISONING.

The first chapter is on the physiological action of poisons, and presents a succinct account of our present state of knowledge on this point. Of the corrosive and irritating effects of some poisons there is ocular demonstration; but of their *nervous impressions*, without any visible organic change, "few well authenticated and unequivocal instances are known." Mr. Brodie mentions a good example in the effects of monk's-hood on the lips when chewed, which causes a sense of numbness and tingling on the lips that lasts for some time, quite unconnected with any affection of the general nervous system. M. Robiquet felt the numbing impression of strong prussic acid even on his fingers. The application of opium and ticunas to the inner surface of the intestines of a rabbit by Drs. Philip, Morgan, and Addison, caused paralysis of the muscular contractions of the gut, without affection of the general system. The same effects have been observed from prussic acid by M. Coullon and others. These facts prove the existence of local impressions of a purely nervous nature, and support the doctrine of the sympathetic operation of poisons against the scepticism of some physiologists.

The influence of a poison can only be conveyed from one organ to another by the sympathetic operation above-mentioned, or by the entrance of the poison into the bibulous vessels, and its mixture with the tide of the circulation. In the infancy of toxicology, all poisons were believed to act by sympathy, or nervous impression:—Magendie's experiments rendered the doctrine of absorption almost universal.

"But the latest researches tend rather, in my opinion, to show that some poisons act by sympathy without entering the blood, and that, although many poisons do enter the blood, the operation even of these, nevertheless, consists of an impression made on the sentient extremities of the nerves and conveyed thence along their filaments to the brain or other organs." 5.

The physician who is in constant attendance at the bed-side of sickness, and who becomes familiar with the phenomena of disease, cannot doubt this reciprocity of influence among the various organs.

There is a large class of poisons, however, whose operation is so slow that absorption has time to take place, and most probably does take place. This subject is handled in a very able manner by our author.

Poisons are believed to act through the blood for the following reasons:—first, they disappear during life from shut cavities—that is, they become absorbed, of which numerous examples are on record, from the experiments of Coindet, Monro, Magendie, Brodie, Barry, and Dr. Christison himself. It has also been ascertained that the absorbing power of the different tissues makes a great difference in the result. The most rapid channel of absorption is from a wound, or by express injection into a vein. The surface of the serous membranes is a less rapid medium, and the mucous membrane less rapid still. The activity of many poisons is in proportion to the absorbing power of these tissues.

"Lastly, of one poison, namely nux vomica, it has been proved, that if the extract be thrust into the paw of an animal after a ligature has been tightened round the leg so as to stop the venous, without stopping the arterial circulation of the limb, blood drawn from an orifice in a vein between the wound and the ligature, and transfused into the vein of another animal, will excite in the latter the usual effects of the poison, so as even to cause death; while, on the contrary, the animal from which the blood has been taken will not be affected at all, if a sufficient quantity is withdrawn before the removal of the ligature." 10.

To these strong arguments, physiologists on the other side of the question have replied, that, the disappearance of a poison from a short sac, as well as other experiments, only prove that poisonous substances do enter the blood, but do not prove that they are carried to the organs on which they act—or that such transmission is absolutely necessary.

"If by the same test (say they) which has been adopted by Vernière, we attempt to prove the existence of the poison in the arterial blood, or in the general blood of the body, our efforts will fail completely. If, for example, the carotid and jugular vein of one dog be divided, and both ends of each reciprocally connected by proper tubes with the divided ends of the carotid and jugular of another dog, and extract of nux vomica be introduced into a wound of the face or neck of one of them, this animal will perish in the usual time, while the other will remain unharmed to the last. This well devised experiment, which is mentioned by *Mr. Morgan* and *Dr. Addison*, in their conjunct inquiry on the action of poisons, proves that the poison is not carried with the blood to the organ which is acted on; although the above-mentioned experiment of Vernière also clearly shows that the poison really enters the veins where it is immediately applied.—The same reply may be given to the famous experiment of Magendie on the effect of the extract of nux vomica, when the part where it has been applied communicates with the body only by means of two tubes, which join together the divided ends of an artery and a vein. The experiment proves that the poison enters the blood and in substance reaches the trunk; but it proves no more. As to the experiment of Emmert it is sufficient to reply, that the maintenance of the circulation is essential to the right discharge of all the functions, and among the rest to the integrity of all the acknowledged functions of the nerves; consequently the non-action of a poison when a ligature is applied on the vessel which supplies the part where the poison lies, is no proof that the poison acts through the blood.—The argument which is drawn from the activity of many poisons corresponding with the absorbing power of the textures to which they are applied, has not been touched on by the supporters of action by sympathy, but will presently be seen to harmonize with that doctrine, according to its newest modification." 11.

Besides these negative arguments, several positive facts, of much weight, have lately been brought forward. One of these has already been alluded to, viz. the admirable experiment of Morgan and Addison, by which it appears that the arterial blood of an animal under the influence of poison, is not poisonous.

"But the same experimentalists have also shewn that if a poison be introduced into a great vein, with certain precautions for preventing its passage towards the heart, it nevertheless will act with unimpaired rapidity. Thus, if the jugular vein of a dog be secured by two temporary ligatures,—be divided between them,—and then re-connected by a tube which contains woorara, we find of course, on removing both ligatures, that the poison quickly begins to act. But it will act with the same quickness if we remove only the ligature farthest from the heart; which is incompatible with the notion that it must be carried with the blood to the brain, the organ that is affected by it.—They have farther shown that the operation of poisons which appear to act through the blood, is not accelerated by introducing them into the artery which supplies the organ acted on. If the counterpart of the former of the two foregoing experiments be performed on the carotid artery instead of the jugular vein, the woorara does not act more rapidly, as we should anticipate, did this poison act through the blood: and, what is still more to the point, the action is not retarded when the poison is introduced in the same way into the femoral artery. On the contrary, in all the three situations, in the carotid artery, jugular vein, and femoral artery, it acts with the same quickness; which is inconceivable if it acted on the brain through the blood, since in the first situation it passes instantly into that organ, and in the last it accomplishes its course only after passing through the whole systemic and pulmonary circulation." 12.

All these contradictory facts are reconciled by the theory of Dr. Addison and Mr. Morgan. They conceive that the sympathetic action of some poisons is unequivocally established by the great rapidity of their effects—and this being admitted, they think it irrational to suppose that other poisons, causing the same or analogous symptoms, operate by absorption.

"In order to explain the continuance of the action of poisons when, as in Magendie's experiment, the part directly poisoned communicates with the trunk by blood-vessels only, or when, as in their own experiment, the poison is introduced into a vein, but prevented from reaching the heart,—they suppose, that, like the other membranous cavities of the body, the inner surface of the vascular system is supplied with an expansion of nervous filaments, on which poisons produce their peculiar impressions, and from which these impressions are communicated along the nerves to remote organs. In order, however, to account also for the correspondence observed between the activity of poisons and the activity of absorption in the several textures to which they are applied, it would be further necessary to maintain, that the nervous expansion of the inner membrane of the blood-vessels is more peculiarly fitted, than the sentient extremities of the nerves elsewhere, for receiving the impressions made by poisonous agents; nay perhaps, that it is the only nervous expansion which possesses that function, except in regard to those poisons which cause evident organic injury, such as inflammation or corrosion. This important doctrine is one towards which Mr. Morgan and Dr. Addison evidently lean, although they have not in their work adopted it explicitly." 12.

Dr. Christison himself thinks that all these ingenious experiments and theories do not contradict the general principle, that the bibulous veins, as the organs of absorption, perform a very material part in the operation of poisons. It remains indisputably established that at least many poisons enter the blood, although it is doubtful or improbable that any pass with the blood to pervade the structure of the organ acted on.

A distinguished American physician, whose name we do not at this moment recollect, argued, some years ago, and supported his arguments by many facts and observations, that all medicines acted in this sympathetic manner on the body and its functions. And certainly if it be proved that poisons generally act through the medium of the nerves rather than of the blood-vessels, it cannot be doubted that medicines operate in the same way.

In respect to physiology, as well as medical jurisprudence, it becomes a matter of great interest to detect poisons in the circulating fluids. Now certain poisons, after being swallowed, have been detected in the blood, secretions, and excretions, the proofs of which we need not here enumerate.

The spirituous odour of the breath in men and animals under the influence of alcohol, is a proof of the presence of that poison throughout the blood. Nevertheless the more general rule is that poisons which appear to enter the blood, cannot be detected either in that fluid or in the animal solids. This may be owing to the minuteness of the quantities travelling the round of the circulation, or to their being thrown off by the various emunctories before death.

Dr. Christison next adverts to the "organs affected by the remote action of poisons." A few of the poisons, such as arsenic and mercury, appear to affect a great number of organs of the body at one and the same time. But much the larger proportion seems to act on one or more organs only, not on the general system. Many appear to act *sympathetically* on the heart alone. The mineral acids do not enter the blood, and all the symptoms they produce, excepting the direct effects of the local injury, are those of depressed action of the heart—great feebleness, fainting, imperceptible pulse, cold extremities. In this way tobacco acts principally on the circulation, and so do the upas antiar, and some other poisons that act also on many other organs at the same time, as arsenic and oxalic acid. Some poisons act strongly, but not solely, on the lungs. In poisoning by tartar emetic, the lungs are commonly inflamed and sometimes hepatized. The same effects have been observed after sublimate. A great number of poisons act on the brain, as evinced by convulsions, giddiness, delirium, paralysis, coma. Post-mortem effects, however, as congestion or extravasation, are rare and equivocal. It is probably true that most poisons that have a range of remote action, act through the medium of the brain. It will not hold good indeed with those that appear to act solely on the heart or spine, as tobacco and strychnia.

The action of poisons is greatly modified by several causes, as quantity, state of aggregation, chemical combination, mixture, differences in tissues, differences in organ, habit and idiosyncrasy. On all these modifying causes Dr. C. makes judicious observations, winding up this chapter with a short section on the treatment of poisoning generally, which consists in the administration of antidotes of two kinds—one of which takes away the deleterious qualities of the poison, by altering the chemical nature—the other controls the poisonous action after it has begun, by exciting a contrary action in the system. Few antidotes of the latter kind are at all satisfactory in their operation, and consequently they are little trusted. It is chiefly to the changes induced among the chemical affinities that the practitioner must look for counter-poisons. The ingenuity of the toxicologist has supplied the materia medica with many of singular efficacy, as magnesia or chalk for the mineral and oxalic acids, albumen for sublimate, bark for tartar emetic, &c. In respect to external poisons, the grand object is to prevent the introduction of the deleterious substance into the circulation, or remove it from the local vessels which it has entered. With both these views, the cupping-glass, as illustrated by Dr. Barry, is the most efficacious antidote. Another mode is a combination of ligature with venesection.

"Suppose a fatal dose of extract of nux vomica has been thrust into the paw of a dog; M. Vernière applies a tight ligature round the limb, then slowly injects as much warm water into the jugular vein as the animal can safely bear, and then slackens the ligature. The state of venous *plethora* thus induced completely suspends absorption. The ligature is next tied so as to compress the veins without compressing the arteries of the limb, and a vein is opened between the wound and the ligature in such a situation, that the blood which flows out must previously pass through the poisoned wound. When a moderate quantity has been withdrawn, the ligature may be removed with safety; and the extraction of the poison may be farther proved by the blood that has been drawn being injected into the veins of another animal, for rapid death by tetanus will be the result. It is not improbable that in this plan the preliminary production of venous plethora may be dispensed with; and then the treatment may be easily and safely applied to the human subject." 29.

We have now arrived at the second chapter of the work, discussing the "Evidence of General Poisoning."

The art of secret poisoning, which once made so much noise in the world, is treated with scepticism by our author. It is not likely that the practices of Toffana or Brinvilliers will ever be renewed. The evidence of the existence or non-existence of poisoning in general (that is, without reference to any particular poison) is derived from five sources—the symptoms, the post-mortem appearances, chemical analysis, experiments on animals—moral circumstances. It will not be uninteresting or useless to touch a little on each of these subjects, especially as they have been but imperfectly investigated by preceding writers.

### I. Evidence from Symptoms.

Not many years ago questions of poisoning were decided by symptoms alone. But no such evidence is now trusted to, per se. Still the symptoms are great auxiliaries, and in some cases, as of mineral acids, arsenic, sublimate, and strychnine, they are almost conclusive. The chief characteristics of the symptoms in poisoning are that they commence suddenly, proceed rapidly to a fatal issue—that they increase steadily—are uniform throughout their course—begin after a meal—and appear when the body is in a state of health. The exceptions to each of these characteristics are very numerous, and so very evident that we shall not dwell on them. Thus the first characteristic is exceedingly liable to exceptions, where the dose is small. Arsenic, digitalis, strychnine, &c. may be introduced into the system slowly, and yet so as to produce their specific and fatal effects at last. Dr. C. makes many judicious observations on the natural diseases which may commence, proceed, and terminate in the way which poisons do, and which, of course, throw doubt on symptomatology taken alone. Thus, for example, with respect to the appearance of symptoms of poisoning "*very soon after a meal*," we know that this is the time at which some of the most fatal disorders commence, as apoplexy, cholera, perforation of the stomach from chronic ulceration, &c. At the same time it is to be recollected that the diseases which commence immediately after food, and proceed to a fatal and sudden termination, are few in number, and none of them by any means frequent. The occurrence of such a circumstance may, therefore, justly be deemed suspicious. The same may be said respecting the other items above enumerated. The following passage is curious and interesting.

" On the other hand, if the symptoms do not begin soon after food, drink, or medicine has been taken (the circumstances being such as to exclude the possibility of poison being introduced by a wound, by the lungs, or by any other channel but the stomach,) the presumption on the whole is against poisoning, and sometimes the evidence to this effect may be decisive. The principle now propounded may be often a very important one in the practice of medical jurisprudence; for when united with a little knowledge of the symptoms antecedent to death, it may be sufficient to decide the nature of the case. Thus it is sufficient, in my opinion, to decide the late celebrated case of the *Crown Prince of Sweden*. The Prince, while in the act of reviewing a body of troops on the 28th May, 1810, was observed suddenly to waver on his horse; and soon afterwards he fell off while at the gallop, was immediately found insensible by his staff, and expired in half an hour. As he was much beloved by the whole nation, a rumour arose that he had been poisoned; and the report took such firm root in the minds of all ranks, that a party of military, while escorting the body to Stockholm, were attacked near the city by the populace, and their commander, Marshall Fersen, murdered; and Dr. Rossi, the Prince's physician, after narrowly escaping the same fate, was in the end obliged to quit his native country. Now, no other poison but one of the most active narcotics could have caused such symptoms, and none of them could have proved so quickly fatal unless given in a large dose. It was proved, however, that on the day of his death the Prince had not taken any thing after he breakfasted; and an interval of nearly four hours elapsed after that till he fell from his horse. This fact alone, independently of the marks of apoplexy found in the head after death, and the warnings he had several times before it, was quite enough to show that he could not have died of poison, as it was incompatible with the known action of the only poisons which could cause the symptoms." 40.

We all know how often an outcry is raised against individuals or a family, when a member happens to die suddenly, the family itself not being in harmony with each other. In several instances of this kind, our author has been induced to dispense with an analysis, by resting on the criterion alluded to in the foregoing extract.

"A middle-aged man, who had long enjoyed excellent health, one afternoon about two o'clock returned home tired, and after having been well beaten by his wife went to bed. At a quarter past two one of his workmen found him gasping, rolling his eyes, and quite insensible; and he died in a few minutes. As his wife had often maltreated and threatened him, a suspicion arose that he had died of poison, and the body was in consequence examined judicially by *Mr. Newbigging* and myself. The only appearance of disease we could detect was a considerable tuberculation of the septum cordis and anterior parietes of both ventricles. This disease might have been the cause of death; for there is no disease of the heart which may not remain long latent, and prove fatal suddenly. But, as the man never had a symptom referrible to disease of the heart, it was impossible to infer, in face of suspicions of poisoning, that it must have been the cause of death; since the man might very well have died of poison, the disease of the heart continuing latent. Poisoning, however, was out of the question. The man had taken nothing whatever after breakfasting about nine. Now, no poison but one of the most active narcotics in a large dose could cause death so rapidly as in this case; and the operation of such a poison in such a dose could not be suspended so long as from nine till two. An analysis was therefore unnecessary." 41.

From the symptoms of poisoning *alone* then, as we said before, no certain criteria can be drawn, though much auxiliary information. We next proceed to the

### EVIDENCE FROM MORBID APPEARANCES.

These, like the former, had great weight in evidence, but often with less reason. Except in the instance of a very few poisons, the morbid appearances *alone*, can never distinguish between the effects of them and of natural disease or violent death. Lividity of the body, and early putrefaction are still thought by the vulgar to be signs of poisoning; though there is no foundation for such an opinion. In poisoning by arsenic, it is well known, that there is the reverse of early putrefaction. The post-mortem appearances are inflammation of the intestinal canal and its products in one class of poisons—congestion of the cerebral vessels in another class—and in a third a combination of the two. But neither set of appearances are invariably caused by the poisons which usually induce them—congestion of the brain is rarely caused by those which are currently supposed to induce them—and, in fine, most of the appearances of both kinds, are exactly similar to those left by many natural diseases. Still the post-mortem appearances are not to be neglected, as they will assist the investigator greatly, in connexion with other species of evidence.

### EVIDENCE FROM CHEMICAL ANALYSIS.

This, of course, is the most decisive of all the branches of proof. It is esteemed most valid when the poison is detected in the stomach, intestines, or œsophagus—then in the matter vomited—and next, in articles of food, drink, or medicine of which the sufferer has partaken—and lastly in articles found in the possession of the prisoner. When poison is found in the stomach, a question may arise whether or not it was the cause of death. On this point our author has brought forward some curious illustrations from two very striking cases. The first occurred not long ago to Dr. Wildberg of Bostock.

"Wildberg was required to examine the body of a girl, who died while her father was in the act of chastising her severely for stealing, and who was believed by all the bye-standers, and by the father himself, to have died of the beating. Accordingly, Wildberg found the marks of many stripes on the arms, shoulders, and back, and under some of the marks blood was extravasated in considerable quantity. But these injuries, though severe, did not appear to him adequate to account for death. He therefore proceeded to examine the cavities; and on opening the stomach he found it very much inflamed, and lined with a white powder, which proved on analysis to be arsenic. It turned out, that on the theft being detected, the girl had taken arsenic for fear of her father's anger, that she vomited during the flogging, and died in slight convulsions. Consequently, Wildberg very properly imputed death to the arsenic. In this case the chemical evidence proved that poison had been taken; but an account of the symptoms and appearances was necessary to prove that she died of it.*—The other case occurred to *Pyl* in 1783. A woman at Berlin, who lived on bad terms with her husband, went to bed in perfect health; but soon afterwards her mother found her breathing very hard, and on inquiring into the cause discovered a wound in the left side of the breast. A surgeon being immediately sent for, the hæmorrhage, which had never been great, was checked without difficulty; but she died nevertheless towards morning. On opening the chest it appeared that the wound pierced into it, and penetrated the pericardium, but did not wound the heart, and although the fifth intercostal artery had been divided, hardly any blood was effused into the cavity of the chest. Coupling these circumstances with the trifling hemorrhage during life, and the fact that she had much vomiting and some convulsions immediately before death, Pyl satisfied himself that

* "*Wildberg*. Praktisches Handbuch für Physiker, iii. 227."

she had not died of the wound; and accordingly the signs of corrosion in the mouth and throat, and of irritation in the stomach, with the subsequent discovery of the remains of some nitric acid in a glass in her room, proved that she had died of poison.*" 49.

We agree with our author that it is almost certain that the real nature of these two deaths would not have been discovered in this country, from the inattention to medico-legal investigations, on the part both of the profession and the bench.

We are next to inquire what are the causes which may remove the poison beyond the reach of the inspector—it may have been discharged by vomiting or purging—absorbed—or decomposed.

In the trial of George Thom for poisoning the Mitchells, on the Aberdeen Circuit, 1821, it was clearly proved that the deceased had died from arsenic, although none could be detected in the stomach, for the man lived seven days, labouring under incessant vomiting. In a case where the patient lived only five hours, Dr. C. could not detect more than a fifteenth part of a grain of arsenic in the contents and coats of the stomach. A case is related in an American Journal, where a man had swallowed an ounce of arsenic, and died in eight hours—yet no poison could be detected by chemical analysis. And yet it is singular how ineffectual vomiting often proves in the expulsion of some poisons from the stomach. Arsenic and others which are not easy of solution may remain adhering to the villous coats, notwithstanding repeated and violent efforts to dislodge them by vomiting.

In respect to absorption, it has several times happened that laudanum, and even solid opium has soon disappeared in bodies poisoned with these substances. Dr. C. and Mr. Newbigging could not detect laudanum in the body of one who undoubtedly swallowed it, and who died in about eight hours. Pyl has related a similar instance. Great errors have been committed by medical witnesses in consequence of overlooking the fact of absorption. Dr. C. alludes to a recent instance which happened at a Coroner's Inquest in London.

"A young man one evening called his fellow-lodger to his bedside; assured him he had taken laudanum, and should be dead by the morrow; and desired him to carry his last farewell to his mother and his mistress. His companion thought he was shamming; but next morning the unfortunate youth was found in the agonies of death. The moral evidence was not very satisfactory; but that is of little consequence to my present object. The point in the case I would particularly refer to is the declaration of the medical inspector, that laudanum could not have been taken, because he did not find any by the smell or by chemical analysis in the contents of the stomach."† 52.

Lastly the excess of a poison may be decomposed. Vegetable and animal substances may be altogether destroyed by the process of digestion. The case of a French soldier is related, who died in six hours and a half after swallowing two drachms of solid opium, none of which could be found after death, nor the smell of opium detected. Some mineral poisons, as sublimate, lunar caustic, hydrochlorate of tin, are also decomposed in the stomach, but they are not removed beyond the reach of chemical analysis. The decomposition is a chemical, not a vital process, and the base

* "Aufsätze und Beobachtungen, &c. ii. 122."
† "Morning Chronicle, Jan. 8, 1823."

of the poison may be found in the solid contents of the stomach under some other compound form. The decomposition of the body also may render it impossible to detect poison that has been actually swallowed. Oxalic acid may be dissolved and then exude—vegetable narcotics may putrefy, and Prussic acid may be volatilized. Arsenic, however, has been detected in the body fourteen months after interment.

### Experiments on Animals.

This is more equivocal than was once imagined; but our author thinks that some medical jurists have overstepped the proper limits in rejecting this species of proof in toto. Still it is not likely to be resorted to directly in cases of poisoning; but the practitioner should be acquainted with the opinions which have been given by others—too rashly no doubt—and which may be canvassed in a court of law. An important objection, indeed to this species of evidence, is the fact that what is poison to man is not always poison to animals, and vice versa. It is curious that the cat and the dog are affected by almost all poisons exactly in the same way as man—the dog more particularly.

"In general poisons act less violently on these animals; thus two drachms of opium are required to kill a middle-sized dog*, while thirty-six grains have killed a man, and probably a much less quantity would be sufficient for the purpose. It appears that one poison, alcohol, acts more powerfully on them than on man. There are also some poisons, such as opium, which, although deleterious to them as well as to man, nevertheless produce in general different symptoms. Yet the differences alluded to are not greater perhaps than exist between man and man in regard to the same substances; and therefore I think it may be assumed, that, on the whole, the effects of poisons on man differ little from those produced on the dog and cat." 55.

On the whole, it appears that, in the present state of our knowlege, experiments or accidental observations on the effects of the contents of the stomach or matters vomited on animals are very equivocal in their import.

"At the same time I must observe, as with regard to articles of food, drink, or medicine, that the effects of some poisons on man may be developed so characteristically on animals by the contents of the stomach, as to supply very pointed evidence indeed. Of the force of this statement the following example is a striking illustration. In the case of a girl, who was proved to have died of accidental poisoning with laudanum, the inspector evaporated the contents of the stomach to dryness, made an alcoholic extract from the residue, and giving this to several dogs, chickens, and frogs, found that they were all made lethargic by it, some of them oftener than once, and that a few died comatose. Facts such as these, agreeing so pointedly with the known effects of the poison suspected, appear to me to yield evidence almost unimpeachable." 60.

A long section, of 13 pages, follows on moral or circumstantial evidence, containing many judicious and ingenious reflections and observations, but such as we cannot analyze. There is no doubt that the medical man, in the course of his professional investigation of the case, has excellent opportunities of acquiring information of the moral and circumstantial kind, which may often be of great advantage both to himself and the court. The heads of moral evidence, to which the medical practitioner ought to direct atten-

* "*Charret*, in Revue Médicale 1827, i. 514."

tion, while proceeding in his more immediately professional course, are the following.

"1. To suspicious conduct on the part of the prisoner before the event, such as dabbling with poisons when he has nothing to do with them in the way of his profession, conversing about them, or otherwise showing a knowlege of their properties not usual in his sphere of life:—2. To the purchase or possession of poison recently before the date of the alleged crime, and the procuring it under false pretences, such as for poisoning rats when there are none on his premises to poison, or for puposes to which it is never applied:—3. To the administration of poison, either in food, drink, or medicine, or otherwise:—4. To the intent of the prisoner, such as the impossibility of his having administered the poison ignorantly, or by accident, or for beneficial purposes, alleged or not alleged:—5. To the fact of other members of the family, besides the deceased, having been similarly and simultaneously affected:—6. To suspicious conduct on the part of the prisoner during the illness of the person poisoned,—such as directly or indirectly preventing medical advice being procured, or the relations of the dying man being sent for, or showing an over-anxiety not to leave him alone with any other person, or attempting to remove or destroy articles of food or drink, or vomited matter which may have contained the poison, or expressing a foreknowledge of the probability of speedy death:—7. To suspicious conduct after the person's death, such as hastening the funeral, preventing or impeding the inspection of the body, giving a false account of the previous illness, showing an acquaintance with the real or supposed effects of poison on the dead body;—8. To the personal circumstances and state of mind of the deceased, his deathbed declaration, and other particulars, especially such as tend to prove the impossibility or improbability of suicide;—9. To the existence of a motive or inducement on the part of the prisoner, such as his having a personal quarrel with the deceased, or a hatred of him,—his succeeding to property by his death, or being relieved of a burden by it,—his knowing that the deceased was with child by him." 62.

On all these points our author makes many sensible commentaries, which we are obliged to pass over, and proceed to the last chapter of this first part of the work.

### Imaginary, Pretended, and Imputed Poisoning.

The leading points to be attended to in this kind of investigation are laid down by our author, and an actual example of each variety is given by way of illustration.

*Imaginary poisoning* can hardly occasion deception or embarrassment. The same hallucination which induces the belief of poison, will betray itself when the investigation bears upon the symptoms, the administration, &c. of the poison. Thus Dr. C. was applied to in a case of this kind lately, where a lady fancied that poison was administered to her. The moral particulars were all plausible enough. Camphor was the vehicle in which the enemy (a relative) had administered the poison; but, unluckily for the consistency of the story, she stated that the poison could only have been given in wine—that the wine had no particular taste—that she did not take ill till the day after drinking the wine—but that camphorous perspiration had exhaled from her skin from that time forward. Her illness turned out to be a slight general fever.

*Feigned or pretended poisoning* is much more apt to escape suspicion—and, when suspected, is difficult to develop satisfactorily, because the actor has it in his power to lay his plans with care, and even to make himself acquainted with the properties of the poison whose effects he intends to feign. Still he can rarely enact his part so well as to deceive a skilful physician.

"In a case of feigned poisoning an excellent mode of investigation is, after hearing out the individual's own story to put a number of questions involving an alternative answer, one alternative being compatible and the other incompatible with the alleged nature of his illness. No unprofessional person could stand such a system of interrogation, if skilfully pursued. Not only will his answers be often wrong; but likewise his manifest perplexity how to answer will of itself supply evidence of his falsehood." 77.

Great attention must also be paid to the chemical analysis. The following case will illustrate many of the rules to be observed on such occasions.

"A young married female, in the seventh month of pregnancy, having been discovered by her friends to be secretly addicted to dram-drinking, appeared to be much annoyed in consequence of the discovery; and one evening was found apparently very ill by her husband on his return from work. She represented that she had taken arsenic with a view to self-destruction, that she was in great torture, and that she was sure she would soon die. It was accordingly found, on reference to a neighbouring apothecary, that she had the same forenoon purchased about a drachm and a half of arsenic, for the alleged purpose of poisoning rats; and in the bottom of a tea-cup, in which she said she mixed it, there was left a small quantity of white powder, that proved on analysis to be pure oxide of arsenic. Notwithstanding these strong facts, the mildness of the symptoms and the composure with which she complained of her tortures led her friends to suspect she was feigning. On investigating her case I first found, in further corroboration of her story, that the powder was nowhere to be found. But she then stated in reply to questions involving an alternative answer, that the arsenic had a sour taste, and that the pain began in the lower part of the belly and spread upwards. She likewise said that she vomited a mouthful or two into a chamber-pot twenty minutes after taking the poison, that she vomited no more till the apothecary was sent for, who gave her emetics of sulphate of zinc, carefully preserving the discharges, and that she only vomited when emetics were given. When I first saw her, five hours after the alleged date of the taking of the arsenic, the skin was warm and moist, the face full and flushed, the pulse frequent and firm, the muscular strength natural. The chamber-pot contained only a small quantity of the fæces of a child and apparently a little water but no matters of vomiting and no white powder. The fluid discharged in presence of the apothecary was found on careful analysis to contain a large quantity of zinc, but not an atom of arsenic. She gradually recovered from the illness under which she laboured at the time I saw her, and in two days she admitted she was quite well, but continued to insist that she had taken the poison." 78.

A remarkable trial for imputed poisoning occurred, not many years ago, at the York assizes, where a man named Whalley, was indicted for administering arsenic to Martha King, who was pregnant by him.

"The woman King swore, that the prisoner, after twice trying, but in vain, to prevail on her to take drugs for the purpose of procuring abortion, sent her a present of tarts, of which she ate one and a half,—that in half an hour she was seized with symptoms of poisoning with some irritant poison,—and that she continued ill for a long time after. Mr. Thackrah found arsenic in the tarts that remained untouched, and likewise in some matter that was vomited in his presence after the administration of an emetic, as well as in other matters of vomiting which were preserved for him between his first and second visits. Her appearance, however, did not correspond with the complaint she made of her sufferings, her pulse and tongue were natural, and on careful investigation the following inconsistencies were farther detected. 1. She said she felt a coppery taste in the act of eating the tarts, a taste which arsenic certainly never possesses.

2. From the quantity of arsenic in the tarts which remained she could not have taken above ten grains, while even after repeated attacks of vomiting, the alleged matter subsequently preserved contained nearly fifteen grains. 3. The first matters of vomiting contained only one grain, while the matter alleged to have been vomited subsequently contained fifteen grains. 4. The time at which these fifteen grains were alleged to have been vomited was not till between two and three hours after the symptoms began; in which case the symptoms would before that time have been violent. The prisoner was acquitted, and the prosecutor and another woman who corroborated her deposition afterwards admitted that they had entered into a conspiracy to impute the crime to him, because he had deserted her on finding that she was too intimate with other men." 80.

This brings us to the close of the first part of Dr. Christison's book; the whole of the second part—indeed almost the whole of the work, being on "individual poisons," many of which we shall notice separately in our Periscopic articles. Even what we have brought forward from this, which is little more than an introductory portion of the performance, must produce a favourable impression respecting its merits on the reader, but an examination of the whole will convince him that this is a standard publication;—one of the most valuable and necessary which he can place in his library—for study in leisure, as well as reference in the hour of emergency, perplexity, doubt—nay, danger.

